# Spinal Paralysis of the Young Carnivora

**Published:** 1882-01

**Authors:** W. A. Conklin

**Affiliations:** Director Central Park Menagerie.


Editor Journal of Comparative Medicine and Surgery:—

I would state in reply to Doctor Todd's communication, published in the last number of the Journal, relating to the spinal paralysis of the young carnivora born in the St. Louis Zoölogical Garden, that the disease is one that superintendents of zoölogical gardens have most frequently to contend with. It is caused in many cases by the cubs not having sufficient mineral matter in the bone, and consequently the animal is not able to use its limbs, thereby causing paralysis. As soon as the young are able to eat meat, I administer phosphates with good results. During the period of gestation I feed the mother with bones of sheep or goat, as these are most easily digested.

The difficulty in distinguishing between the sexes of the spotted hyæna is owing to the fact that the flesh in front of the clitoris of the female hangs underneath the belly, presenting an appearance similar to the penis of the male; but the matter can be settled if the tail is raised, as the testicles of the male are then visible.

W. A. Conklin, Ph. D.

*Director Central Park Menagerie.*

